# 阿伐替尼治疗伴KIT突变RUNX1::RUNX1T1阳性儿童急性髓系白血病的疗效与安全性分析

**DOI:** 10.3760/cma.j.cn121090-20251027-00482

**Published:** 2026-04

**Authors:** 方圆 郑, 明明 丁, 爱东 陆, 月萍 贾, 慧敏 曾, 乐萍 张

**Affiliations:** 北京大学人民医院儿科，北京 100044 Department of Pediatrics, Peking University People's Hospital, Beijing 100044, China

**Keywords:** 阿伐替尼, KIT突变, RUNX1::RUNX1T1融合基因, 儿童, 白血病，髓系，急性, Avapritinib, KIT mutation, RUNX1::RUNX1T1 fusion gene, Children, Leukemia, myeloid, acute

## Abstract

**目的:**

探讨阿伐替尼治疗伴KIT突变的RUNX1::RUNX1T1阳性儿童急性髓系白血病（AML）的疗效和安全性。

**方法:**

收集2022年9月至2025年6月在北京大学人民医院儿科接受阿伐替尼治疗的伴KIT突变的RUNX1::RUNX1T1阳性AML患儿病例资料，回顾性分析阿伐替尼治疗总反应率（ORR）、无事件生存（EFS）率、总生存（OS）率以及不良反应。

**结果:**

10例AML患儿中，男6例，女4例，开始应用阿伐替尼时中位年龄6.5岁。KIT突变类型包括D816V突变8例，D816H突变1例，D816Y突变1例。所有患儿均接受≥1个周期阿伐替尼治疗，其中9例患儿接受≥2个周期阿伐替尼治疗，6例患儿接受≥3个周期阿伐替尼治疗。第1周期后，10例患儿对阿伐替尼治疗的ORR为80％（8/10），其中5例患儿RUNX1::RUNX1T1融合基因水平下降≥1 log；第2周期后ORR为78％（7/9），第3周期后ORR为67％（4/6）。中位随访时间10个月，EFS率为90％，OS率为100％。10例患儿在阿伐替尼治疗期间有5例RUNX1::RUNX1T1融合基因水平升高。阿伐替尼联合化疗期间的不良反应大多与化疗药物有关，表现为血液学不良反应以及胃肠道反应、感染等，均对症处理后好转。在单药或联合干扰素治疗期间以1～2级血液学不良反应为主，其中1例出现惊厥，停药对症处理后好转。

**结论:**

阿伐替尼治疗伴KIT突变的RUNX1::RUNX1T1阳性AML患儿初期具有良好的反应率，能够获得更深层次的分子学缓解，但随着治疗时间延长，反应率下降。阿伐替尼治疗具有较高的安全性。

## Abstract (en)

**Objective:**

To evaluate the efficacy and safety of avapritinib in children with RUNX1::RUNX1T1-positive acute myeloid leukemia（AML）with KIT mutations.

**Methods:**

Clinical data on children with RUNX1::RUNX1T1-positive AML with KIT mutations who received avapritinib at the Department of Pediatrics, Peking University People's Hospital, from September 2022 to June 2025 were collected. The overall response rate（ORR）, event-free survival（EFS）, overall survival（OS）, and adverse reactions of avapritinib treatment were retrospectively analyzed.

**Results:**

Ten children（6 males, 4 females）with a median age of 6.5 years were enrolled. All carried KIT mutations（D816V, *n*＝8; D816H, *n*＝1; D816Y, *n*＝1）. All children completed ≥1 cycle of avapritinib; 9 received ≥2 cycles, and 6 received ≥3 cycles. After cycle 1, the ORR was 80％（8/10）, with 5 children achieving ≥1 log reduction in RUNX1::RUNX1T1. The ORR was 78％（7/9）after cycle 2 and 67％（4/6）after cycle 3. With a median follow-up of 10 months, the EFS rate were 90％, and the OS rate was 100％. During avapritinib treatment, 5 of the 10 children showed elevated levels of the RUNX1::RUNX1T1. During combination avapritinib and chemotherapy, adverse events were largely attributable to chemotherapy, manifesting as hematological adverse reactions, gastrointestinal reactions, and infections, all of which improved after symptomatic treatment. When avapritinib was used alone or with interferon, toxicity was mainly associated with grade 1–2 hematological adverse reactions; one case developed convulsions and improved after drug withdrawal and symptomatic treatment.

**Conclusion:**

Avapritinib provides a favorable initial response in children with RUNX1::RUNX1T1-positive AML harboring KIT mutations, enabling deeper molecular remission; however, response rates decline with prolonged treatment. Avapritinib has a favorable safety profile.

t（8;21）（q22;q22）是急性髓系白血病（AML）患者中常见的染色体异常，产生RUNX1::RUNX1T1融合基因，儿童AML中该融合基因阳性率为15％～20％，阳性患者虽对传统化疗反应良好，但如果合并KIT突变，复发风险骤升[1]–[4]。体外实验显示细胞发生KIT突变后活化速度大幅升高，尤其发生KIT D816V突变后，活化速度可增加536倍，持续激活下游信号通路，导致肿瘤细胞恶性增殖[5]。因此，靶向抑制KIT突变是改善RUNX1::RUNX1T1融合基因阳性患者预后的重要思路[6]。高选择性KIT抑制剂阿伐替尼在成人研究中已显示良好效果，其可迅速降低骨髓原始细胞并清除微小残留病（MRD），桥接异基因造血干细胞移植（allo-HSCT）后获得持续分子学缓解[7]。基于此，本中心在伴有KIT突变的RUNX1::RUNX1T1阳性AML患儿中使用阿伐替尼，评估该药的疗效与安全性，旨在为该高危亚组的精准分层治疗提供循证依据，现报道如下。

## 病例与方法

1. 病例资料：回顾性分析2022年9月至2025年6月在北京大学人民医院儿科住院的AML患儿的临床资料。所有患者的诊断均符合文献[8]标准。纳入标准：①骨髓标本分子学提示同时存在KIT突变阳性和RUNX1::RUNX1T1融合基因阳性；②应用阿伐替尼治疗。

本研究方案已获得医院伦理委员会批准（批件号：2025PHB324-001），所有患儿的监护人均在治疗前被充分告知治疗方案，并签署知情同意书。

2. 阿伐替尼用药方案：阿伐替尼用于化疗间歇期时，单药或联合干扰素治疗。阿伐替尼具体口服剂量参照文献[9]–[10]及成人推荐剂量（300 mg/次）酌减，每日1次，至少在餐前1 h和餐后2 h空腹给药，连续应用3周。用药期间每周监测血常规、肝肾功能等。视耐受性及化验结果适当调整药物剂量和疗程。

阿伐替尼用于联合化疗时，剂量同上，联合化疗用药时间为1周，联合化疗方案参考《儿童急性髓系白血病诊疗专家共识（2024）》[8]及《中国复发难治性急性髓系白血病诊疗指南（2023年版）》[11]。

3. 资料收集及随访：收集患儿基本信息及治疗情况，包括性别、年龄、病程、其他共突变情况、既往靶向药种类、阿伐替尼治疗方案、阿伐替尼治疗前后骨髓情况、髓外浸润情况、后续主要治疗方案等。

随访时间从阿伐替尼开始治疗时间计算。本研究的随访截止日期为2025年9月30日，随访资料来源于住院和门诊病历及电话随访记录。

4. 疗效评估及其定义：所有患儿均在应用阿伐替尼后每1.5～2个月进行骨髓评估，内容包括：①骨髓细胞形态学；②采用多参数流式细胞术（FCM）检测MRD；③采用实时定量聚合酶链反应（RQ-PCR）检测RUNX1::RUNX1T1融合基因水平。疗效定义：①完全缓解（CR）：骨髓原始细胞比例<5％，外周血中性粒细胞≥1×10^9^/L，PLT≥100×10^9^/L，且无髓外浸润；②MRD阴性：符合CR标准，且FCM检测原始细胞低于检测值（即<0.01％）；③RUNX1::RUNX1T1融合基因阴性：符合CR标准，且RQ-PCR检测RUNX1::RUNX1T1融合基因低于检测值（即<0.0001％）。治疗反应：①骨髓形态学CR，且MRD或RUNX1::RUNX1T1融合基因水平低于治疗前水平，且无髓外病灶进展，判定为治疗有反应；②骨髓形态学未达CR，或MRD和（或）RUNX1::RUNX1T1融合基因水平高于治疗前基线水平，或出现髓外病灶进展，判定为治疗无反应。总反应率（ORR）定义为治疗有反应的例数占总例数的百分比。无事件生存（EFS）时间定义为从阿伐替尼治疗开始至复发、死亡或随访截止的时间。总生存（OS）时间定义为从阿伐替尼治疗开始至任何原因死亡或随访截止的时间。

5. 安全性评估：不良反应中血液学不良反应及非血液学不良反应依据美国卫生及公共服务部常见不良事件评价5.0（CTCAE5.0）标准[12]。

6. 统计学处理：应用SPSS19.0软件进行统计分析。计量资料使用*M*（范围）表示，计数资料以例数（百分比）表示。

## 结果

1. 一般临床资料：本研究共纳入10例患儿，男6例，女4例，开始应用阿伐替尼中位年龄6.5（3～15）岁。KIT突变类型：D816V突变8例，D816H突变1例，D816Y突变1例。所有患儿阿伐替尼治疗前均无髓外浸润。诱导治疗期即开始应用阿伐替尼2例，巩固治疗期开始应用阿伐替尼8例。自诊断到应用阿伐替尼中位间隔时间6.5（0.5～9）个月。10例患儿的临床特征及阿伐替尼使用情况见表1。

**表1:** 10例伴有KIT突变的RUNX1::RUNX1T1阳性儿童急性髓系白血病患者的临床特征及阿伐替尼使用情况

例号	性别	年龄（岁）	KIT突变类型	既往靶向药种类	阿伐替尼应用原因	阿伐替尼应用前MRD、RUNX1::RUNX1T1融合基因表达	阿伐替尼应用方案
1	女	4	D816V	达沙替尼	基因升高	MRD阴性、基因定量0.017％	①HDAra-C方案，后阿伐替尼×3周
						MRD阴性、基因定量0.0014％	②CLAG方案，后阿伐替尼×3周
						MRD阴性、基因定量0.0079％	③CAG方案，后阿伐替尼×3周
2	男	5	D816V	达沙替尼	基因升高	MRD阴性、基因定量0.023％	①CLAG方案，后阿伐替尼×3周+干扰素
						MRD阴性、基因定量0.034％	②CAG方案，后阿伐替尼×3周+干扰素
						MRD阴性、基因定量0.0068％	③CAG方案，后阿伐替尼×3周+干扰素
3	男	6	D816V	达沙替尼	基因升高	MRD阴性、基因定量1.401％	①CLAG方案，后阿伐替尼×3周
						MRD阴性、基因定量1％	②CAG方案+阿伐替尼×1周，后阿伐替尼×3周
4	男	7	D816V	达沙替尼	基因升高	MRD阴性、基因定量0.21％	①CAG方案，后阿伐替尼×3周+干扰素
						MRD阴性、基因定量阴性	②HA方案+阿伐替尼×1周
						MRD阴性、基因定量阴性	③HDAra-C方案+阿伐替尼×1周，后阿伐替尼×3周
5	女	9	D816V	达沙替尼	基因升高	MRD阴性、基因定量0.12％	①CLAG方案，后阿伐替尼×3周
						MRD阴性、基因定量0.0058％	②CAG方案+阿伐替尼×1周，后阿伐替尼×3周
6	女	3	D816Y	初诊即选择	诱导期应用	MRD 71.78％、基因定量361.55％	①DAE方案+阿伐替尼×1周
						MRD3.59％、基因定量99.08％	②DAE方案+阿伐替尼×1周
						MRD阴性、基因定量1.35％	③HDAra-C方案，后阿伐替尼×3周+干扰素
						MRD阴性、基因定量0.59％	④DAE方案+阿伐替尼×1周
7	男	6	D816V	达沙替尼	基因升高	MRD阴性、基因定量0.11％	①CAG方案+阿伐替尼×1周，后阿伐替尼×3周+干扰素
						MRD阴性、基因定量0.041％	②CLAG方案，后阿伐替尼×3周
8	男	15	D816V	达沙替尼	基因升高	MRD阴性、基因定量0.22％	①AV方案+阿伐替尼×1周，后阿伐替尼×3周
9	女	10	D816H	达沙替尼	便血	MRD阴性、基因定量0.011％	①HA方案，后阿伐替尼×3周+干扰素
						MRD阴性、基因定量阴性	②HDAra-C方案，后阿伐替尼×3周+干扰素
						MRD阴性、基因定量阴性	③HA方案，后阿伐替尼×3周+干扰素
10	男	12	D816V	初诊即选择	诱导期应用	MRD 28.7％、基因定量361.98％	①DAE方案+阿伐替尼×1周
						MRD 0.1％、基因定量2.93％	②DAE方案+阿伐替尼×1周
						MRD阴性、基因定量2.6％	③HDAra-C方案，后阿伐替尼×3周

**Table:** 

例号	性别	年龄（岁）	阿伐替尼应用后MRD、RUNX1::RUNX1T1融合基因表达	后续主要治疗方案	随访时间、状态及RUNX1::RUNX1T1融合基因水平
1	女	4	MRD阴性、基因定量0.0014％	化疗、HSCT	随访28个月，存活，基因定量阴性
			MRD阴性、基因定量0.0079％		
			MRD阴性、基因定量0.011％		
2	男	5	MRD阴性、基因定量0.034％	化疗、米哚妥林	随访25个月，存活，基因定量阴性
			MRD阴性、基因定量0.0068％		
			MRD阴性、基因定量0.022％		
3	男	6	MRD阴性、基因定量1％	化疗、HSCT	随访16个月，存活，基因定量阴性
			MRD阴性、基因定量1.8％		
4	男	7	MRD阴性、基因定量阴性	阿伐替尼+干扰素维持	随访11个月，存活，基因定量阴性
			MRD阴性、基因定量阴性		
			MRD阴性、基因定量阴性		
5	女	9	MRD阴性、基因定量0.0058％	化疗、阿伐替尼	随访4个月，存活，基因定量0.0032％
			MRD阴性、基因定量0.0017％		
6	女	3	MRD3.59％、基因定量99.08％	HSCT	随访10个月，存活，基因定量阴性
			MRD阴性、基因定量1.35％		
			MRD阴性、基因定量0.59％		
			MRD阴性、基因定量0.29％		
7	男	6	MRD阴性、基因定量0.041％	化疗、阿伐替尼	随访4个月，存活，基因定量0.0072％
			MRD阴性、基因定量0.0072％		
8	男	15	MRD阴性、基因定量0.40％	HSCT	随访9个月，存活，基因定量阴性
9	女	10	MRD阴性、基因定量阴性	阿伐替尼+干扰素维持	随访10个月，存活，基因定量阴性
			MRD阴性、基因定量阴性		
			MRD阴性、基因定量阴性		
10	男	12	MRD0.1％、基因定量2.93％	化疗、阿伐替尼	随访5个月，存活，基因定量1.1％
			MRD阴性、基因定量2.6％		
			MRD阴性、基因定量1.1％		

**注** MRD：微小残留病；HDAra-C：大剂量阿糖胞苷；CLAG：克拉屈滨、阿糖胞苷、G-CSF；CAG：阿克拉霉素、阿糖胞苷、G-CSF；HA：高三尖杉酯碱、阿糖胞苷；DAE：柔红霉素、阿糖胞苷、依托泊苷；AV：阿糖胞苷、维奈克拉；HSCT：造血干细胞移植

2. 治疗反应：10例患儿均接受≥1个周期阿伐替尼治疗，其中9例患儿接受≥2个周期阿伐替尼，6例患儿接受≥3个周期阿伐替尼。结果表明，第1周期后，10例患儿对阿伐替尼反应良好，ORR为80％（8/10），其中5例患儿RUNX1::RUNX1T1融合基因水平下降≥1 log。第2周期后ORR为78％（7/9），在第3周期后，ORR为67％（4/6）。随访截止时7例（70％）患者RUNX1::RUNX1T1融合基因阴性。

3. 转归：后续治疗中，停用阿伐替尼5例，停药原因均为检测到RUNX1::RUNX1T1融合基因升高，后续行HSCT治疗4例，调整为米哚妥林联合化疗治疗1例。持续应用阿伐替尼的5例中，随访截止时阿伐替尼联合化疗巩固治疗中3例，阿伐替尼联合干扰素维持治疗中2例。

10例患儿中位随访10（4～28）个月，所有患儿均未出现髓外浸润，均为存活状态，EFS率为90％，OS率为100％。

4. 安全性评估：在阿伐替尼联合化疗的12例次中，在治疗期间或治疗后均出现3～4级血液学不良反应。非血液学不良反应包括：①均出现以恶心为主的胃肠道反应，对症治疗后好转；②出现感染12例次（包括呼吸道感染6例次、齿龈感染3例次、皮肤感染2例次、金黄色葡萄球菌败血症1例次），抗感染治疗后好转；③出现眼睑水肿3例。

在阿伐替尼单药或联合干扰素治疗中，治疗期间出现1～2级血液学不良反应，联合干扰素者出现发热（未应用抗生素可自行下降，故考虑为干扰素药物所致）。此外，部分患儿出现眼睑水肿、肝酶轻度升高、皮疹、乏力、肌肉酸痛、多汗。此外，例3在阿伐替尼单药治疗期间出现惊厥1次，头颅CT未见颅内出血，停用阿伐替尼并对症治疗后好转，未出现认知受损，后重启常规化疗药物并行HSCT治疗，患儿未再出现惊厥发作。

## 讨论

阿伐替尼是一种靶向KIT突变的新型酪氨酸激酶抑制剂，对KIT等的活化环区域突变具有高选择活性，其可与细胞内结构域的活性激酶构象结合，从而抑制激酶活化，抑制肿瘤信号转导[13]。本研究结果显示，阿伐替尼对于伴KIT突变的RUNX1::RUNX1T1融合基因阳性儿童AML具有良好的治疗反应，可以获得更深度的分子学缓解。

既往多项报道阿伐替尼在伴KIT突变的AML治疗中疗效显著。Xue等[10]报道7例伴有t（8;21）/KIT突变的AML儿童患者，在allo-HSCT后血液学或分子学复发时接受阿伐替尼单药或联合挽救治疗，在治疗中位时间28 d时获得CR，并且5例RUNX1::RUNX1T1融合基因下降≥2 log，其中4例转阴。我国报道一项对伴KIT突变的复发/难治（R/R）或MRD阳性患者应用阿伐替尼治疗的多中心研究[14]，28例MRD阳性患者中，完成3个周期者MRD转阴率50％；12例R/R患者中，总缓解率为50％，复合CR率为41.7％，部分患者成功桥接allo-HSCT后达MRD阴性。Wang等[15]报道4例伴KIT D816突变的RUNX1::RUNX1T1融合基因阳性移植后复发/MRD阳性患儿，在接受阿伐替尼单药或联合治疗后，KIT突变及融合基因均转阴。Kong等[16]研究发现阿伐替尼对KIT基因D816位点突变与非D816位点突变的疗效相近，治疗3个月时两组MRD转阴率分别为50.0％和66.7％。Getz等[17]报道1例经多线治疗的伴有KIT D816H突变的t（8;21）AML患者，行HSCT后出现复发，在接受阿伐替尼单药治疗后迅速获得深度缓解。本研究中，阿伐替尼疗效明显优于上述研究，可能与本研究样本量小、随访时间短有关，也与联合化疗方案的优化、HSCT及早实施等有关。此外，也与本研究疗效判定标准更宽松（MRD或RUNX1::RUNX1T1融合基因水平下降即视为有效，而非≥1 log），且本研究中患儿未达R/R的标准，可能出现更好的治疗反应，也提示对于KIT突变的患者进行阿伐替尼抢先治疗更能获益。

此外，本研究结果显示，阿伐替尼早期缓解率虽高，却随治疗时间延长而有所降低。一方面需考虑联合化疗方案对原发性疾病的治疗作用，另一方面亦需警惕阿伐替尼耐药的可能。既往体外研究提示[18]，KIT T670I“守门人”突变可通过诱导磷酸结合环远端构象变化，间接削弱阿伐替尼结合介导耐药，显著降低其敏感性。因此，需要延长随访时间，以明确阿伐替尼是否会发生获得性耐药，并对疑似耐药患者开展KIT T670I突变检测。

阿伐替尼最常见的血液学不良反应包括中性粒细胞减少、贫血和血小板减少[15]。非血液学不良事件为眶周/面部水肿、腹泻、恶心、纳差、高胆红素血症、流泪增加，以及颅内出血、认知功能障碍和眼部异常。关于认知功能障碍，年龄越大、阿伐替尼剂量越大，发生率越高[19]–[20]。本研究中在联合化疗期间观察到的不良反应大多与化疗药物有关，而在单药或联合干扰素治疗期间，血液学不良反应较轻，非血液不良反应中出现较多的为眼睑水肿、皮疹、乏力、肌肉酸痛，但均为低级别，通过对症处理后好转。但值得注意的是，本研究中出现惊厥发作1例，影像学检查结果正常，经积极对症处理后该患儿惊厥发作完全缓解，未遗留认知功能损害。结合阿伐替尼可透过血脑屏障，考虑为药物相关神经毒性。因此，建议在进行阿伐替尼治疗期间，需要把握好剂量，条件允许下进行血药浓度监测以便于更好控制不良反应。

总之，本研究初步证实，阿伐替尼对于伴有KIT突变的RUNX1::RUNX1T1阳性AML患儿是一种疗效良好且安全性较高的治疗选择。本研究存在一定局限性：首先，研究为单中心小样本病例系列，入组患儿数量有限，随访时间短；其次，阿伐替尼联合化疗方案并不完全统一，可能存在化疗方案差异对治疗效果的影响；第三，研究过程中未对KIT定量动态监测。此外，早期分子学应答率高，但随治疗延长部分患儿出现分子学反弹，该现象尚未被系统量化。因此，未来仍需要开展多中心、大样本、前瞻性的临床研究，以进一步验证阿伐替尼在该类患儿中的长期疗效与安全性，并探索后期耐药机制。
